# Metagenomic, Metabolomic, and Functional Evaluation of Kimchi Broth Treated with Light-Emitting Diodes (LEDs)

**DOI:** 10.3390/metabo11080472

**Published:** 2021-07-22

**Authors:** Yeong-Ji Oh, Ye-Rin Park, Jungil Hong, Do-Yup Lee

**Affiliations:** 1 Center for Food and Bioconvergence, Department of Food and Animal Biotechnology, Research Institute for Agriculture and Life Sciences, Seoul National University, Seoul 08826, Korea; yerinpark@snu.ac.kr; 2 Division of Applied Food System, College of Natural Science, Seoul Women’s University, 621 Hwarangro, Nowon-gu, Seoul 01797, Korea; hjil@swu.ac.kr

**Keywords:** kimchi, light emitting diode (LED), microbial community, metabolomics, cellular toxicity

## Abstract

The light-emitting diode (LED) has been widely used in the food industry, and its application has been focused on microbial sterilization, specifically using blue-LED. The investigation has been recently extended to characterize the biotic and abiotic (photodynamic) effects of different wavelengths. Here, we investigated LED effects on kimchi fermentation. Kimchi broths were treated with three different colored-LEDs (red, green, and blue) or kept in the dark as a control. Multiomics was applied to evaluate the microbial taxonomic composition using 16S rRNA gene amplicon sequencing, and the metabolomic profiles were determined using liquid chromatography–Orbitrap mass spectrometry. Cell viability was tested to determine the potential cytotoxicity of the LED-treated kimchi broths. First, the amplicon sequencing data showed substantial changes in taxonomic composition at the family and genus levels according to incubation (initial condition vs. all other groups). The differences among the treated groups (red-LED (RLED), green-LED (GLED), blue-LED (BLED), and dark condition) were marginal. The relative abundance of *Weissella* was decreased in all treated groups compared to that of the initial condition, which coincided with the decreased composition of *Lactobacillus*. Compositional changes were relatively high in the GLED group. Subsequent metabolomic analysis indicated a unique metabolic phenotype instigated by different LED treatments, which led to the identification of the LED treatment-specific and common compounds (e.g., luteolin, 6-methylquinoline, 2-hydroxycinnamic acid, and 9-HODE). These results indicate that different LED wavelengths induce characteristic alterations in the microbial composition and metabolomic content, which may have applications in food processing and storage with the aim of improving nutritional quality and the safety of food.

## 1. Introduction

Increasing attention has been paid to various functional foods, including kimchi, which is expected to improve health status and prevent diseases (e.g., COVID-19). Kimchi, a representative fermentation food in Korea, has been globalized owing to its association with beneficial bioactivities such as anti-obesity [1,2,3], anti-arteriosclerosis [4], anti-aging [5,6], and anti-mutagenesis activities [7,8]. Kimchi consists of mixed ingredients including various types of vegetables (e.g., cabbage) as its main ingredient and subsidiary flavoring ingredients (e.g., red pepper powder). Additionally, the quality and characteristics of kimchi are determined by the microbial community originating from vegetables and subsidiary materials. Among the kimchi microbial community, probiotics are dominant, specifically, the following three genera: *Lactobacillus*, *Leuconostoc*, and *Weissella* [9,10,11,12]. Therefore, by changing these multiple factors, different combinations of taste and flavor can be acquired, forming various types of fermented kimchi to meet diverse consumer preferences. Moreover, people prefer commercially available kimchi more than making their own, owing to lifestyle changes (e.g., increases in numbers of single-person households) according to the Korea Agro-Fisheries & Food Trade Corporation (2019). As commercial kimchi is manufactured through natural fermentation without using a separate sterilization process, specific care should be taken for raw material management, and proper storage methods should be used to control undesired fermentation by other types of microorganisms.

Recently, light-emitting diode (LED) technology has gained increasing attention in pharmaceuticals, medicine, and cosmetics in the area of photodynamic therapy (PDT) and photo-biomodulation (PBM) [13]. PDT, which requires non-toxic photosensitizers, light with an appropriate wavelength, and oxygen, is a minimally invasive therapeutic technique applied to the treatment of various medical and surgical conditions [14] (e.g., dermatological disease, skin lesion, hair loss, cancer) and has been successfully applied in the field of dermatology, oncology, microbiology [15,16]. Antimicrobial photodynamic therapy (APDT) exhibits a broad spectrum of activity against target pathogens, such as bacteria, fungi, and viruses [17]. In particular, blue-LED (BLED) or green-LED (GLED) treatments have been studied as a photo-inactivation system for inhibiting the growth of pathogenic microorganisms [18,19]. Bacterial photo-inactivation by LED light was induced based on the presence of photosensitizer compounds (endogenous or exogenous), light, oxygen, and reactive oxygen species (ROS) such as hydroxyl radical, singlet oxygen, and superoxide radical, which are generated by the reaction of the excited photosensitizers. These generated radicals cause cellular damage leading to bacterial death [20]. In the meanwhile, PBM, known as low-level laser light therapy (without any addition of photosensitizing agents), is commonly used to heal or regenerate, stimulate damaged or dying tissues; the opposite of PDT, which tends to kill cells [13,21]. Especially, LED-mediated photobiomodulation (LPBM) has proven to be effective for wound healing effects and treatment of skin fibrosis [22,23], recovery from retinal injury and other ocular diseases [24], relieving pain and inflammation in mice [25], inhibition of cell carcinoma progression [26]. In addition, a few studies show that microbiome composition in humans can be influenced by the application of PBM. Ailioaie & Litscher (2021) [27] demonstrated that infrared (808 nm) light treatment increased the content of *Allobaculum* bacterium, which is associated with a healthy microbiome after 14 days of treatment. PBM therapy at 630 nm and 730 nm significantly altered the diversity and abundance of intestinal flora, reversing the typical increase of *Helicobacter* and uncultured *Bacteroidales*, and the decreasing the *Rikenella* seen in mice [28]. Thus, in the present study, we investigated the possibility that microbiota composition in fermented foods containing live microorganisms can be photomodulated by LED light sources.

With advantages including low power composition and wavelength specificity [29,30], LEDs have been adopted for food processing and storage (e.g., in market display units). Several studies have reported the inhibitory effects of BLED treatment on food spoilage microorganisms [31,32,33]; however, few studies have focused on the effect of LED treatment on the microbial community, which is a critical determinant for product quality, in food fermentation processes. Therefore, the aim of this study was to analyze the characteristic changes in the microbial community and the consequent changes in the metabolomic composition of kimchi induced by LED treatment using three different sources (blue, green, and red). Our findings can provide a foundation for developing new technologies that enhance nutritional quality, improve the hygiene of kimchi, and precisely control the fermentation process.

## 2. Results and Discussion

### 2.1. Compositional Characteristics of Kimchi Microbiota Treated Using Different Colors of LED Treatment

**Figure 1:**
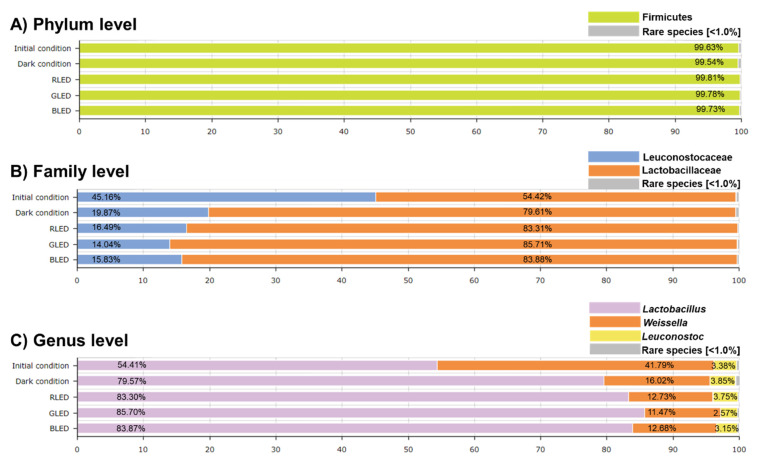
Characteristic changes in microbial taxonomic composition by LED-irradiation at the phylum (**A**), family (**B**), and genus levels (**C**). Firmicutes are the major phylum in all conditions. Leuconostocaceae and Lactobacillaceae are the major family, while *Lactobacillus*, *Weissella*, and *Leuconostoc* are the most dominant genus. Rare species were defined as species found at a frequency of <1% in each sample.

The average of Good’s coverage for all the samples was greater than 99.9%, demonstrating the acceptable level of sequencing depth for bacterial-compositional analysis (Table S1). We first evaluated alpha diversity via Ace, Chao, Jackknife, Shannon, and Simpson indexes based on the results of incubation with different LED treatments (72 h) (Table S1). Samples under the initial condition and no-LED treatment (i.e., incubation in the dark for 72 h) were included as controls. Overall, the microbial diversity (based on Shannon index) decreased with increasing fermentation period (initial condition vs. other groups), which is a typical pattern of the kimchi fermentation process [34,35]. Chao1 index showed the lowest value in the green LED-treated (GLED-treated) kimchi extract, which may be translated to the increased influence on rare species [36]. All tested groups showed the highest abundance of Firmicutes (99.54–99.81%) at the phylum level (Figure 1A). The differences in microbial composition were identified among the tested groups at the family and genus levels (Figure 1B,C). The composition of the Lactobacillaceae family was relatively higher than that of the Leuconostocaceae family in all tested groups, which increased substantially following the 72 h incubation. Likewise, the dominance of *Lactobacillus* genus increased significantly following the incubation and was further moderately raised by all LED treatments (Red-LED (RLED), GLED, and BLEDs). These changes coincided with the decreased dominance in other genera, especially *Weissella*. Few studies have been focused on LED effects on non-pathogenic bacteria, more resistant to an LED treatment compared to pathogenic bacteria [37]. To the best of our knowledge, it is the first report on the LED-induced alteration of probiotics, including *Lactobacillus*, *Weissella*, and *Leuconostoc* in kimchi extract.

### 2.2. Metabolomic Modulation Triggered by Different Colors of LED Treatment

**Figure 2:**
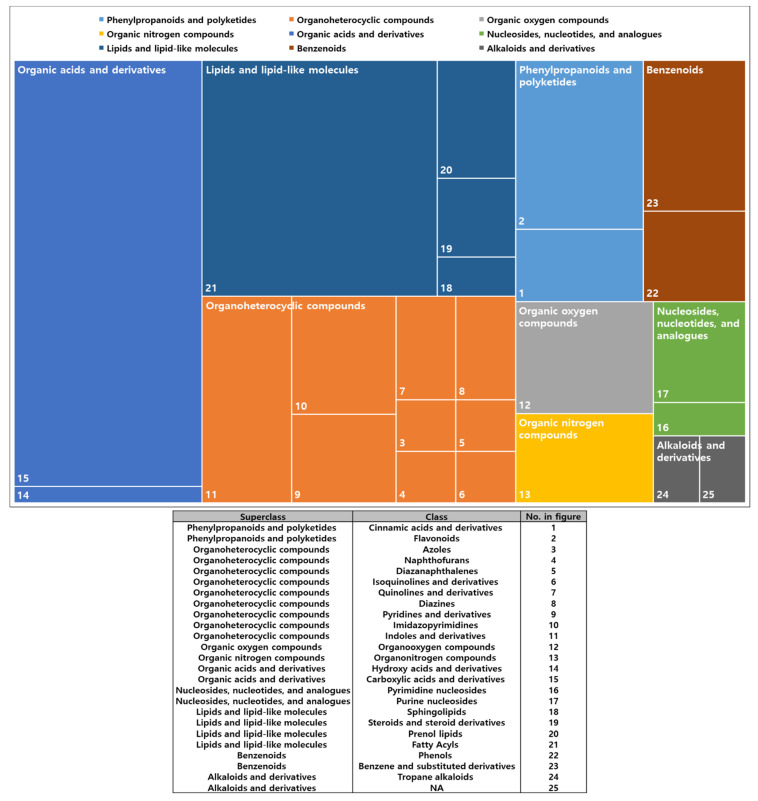
Chemical classification of metabolites from kimchi broths. The classification was done by chemical taxonomy from the Human Metabolome Database (HMDB). Color indicates the same superclass. Each superclass is further divided into class as presented in the box.

**Figure 3:**
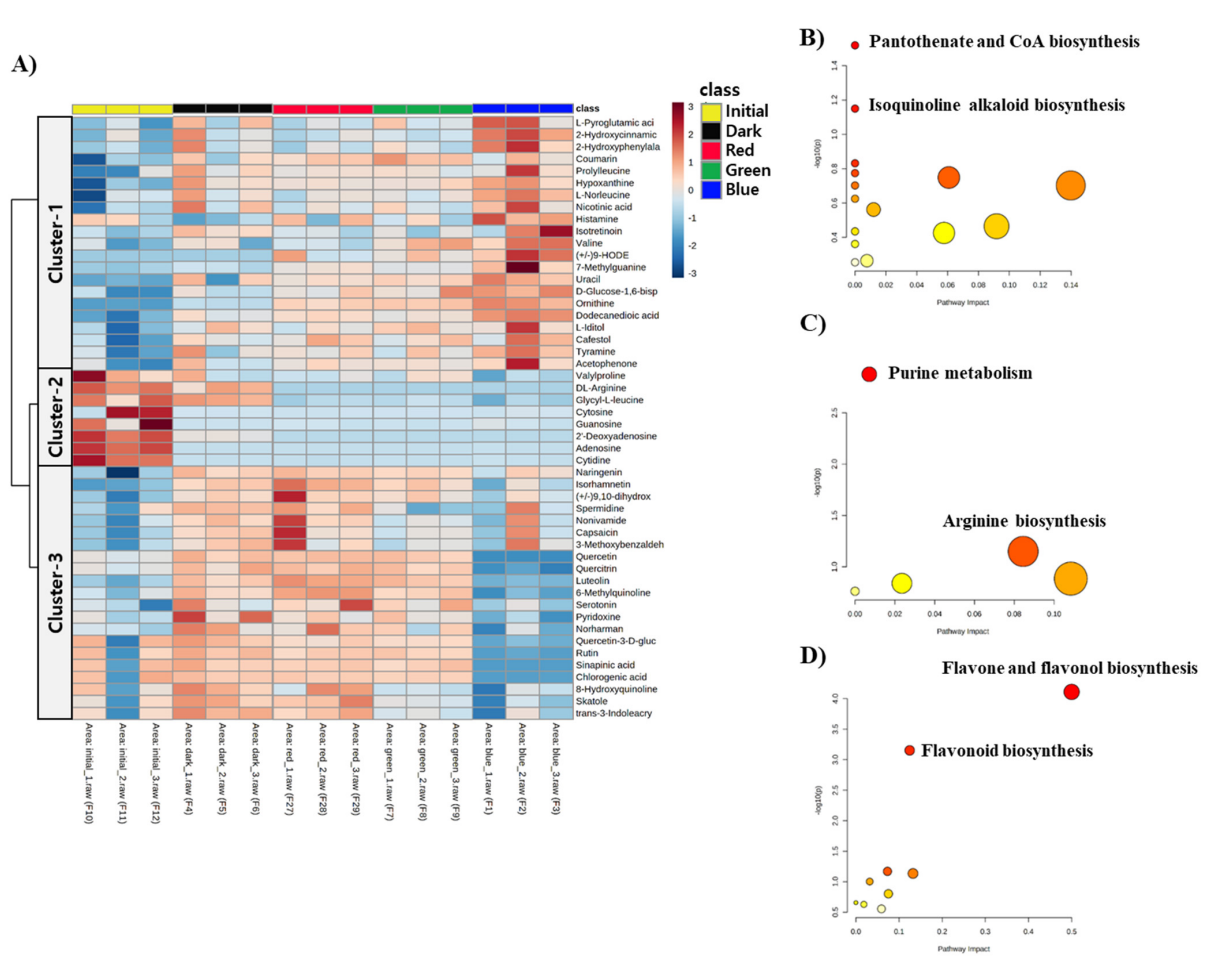
(**A**) Hierarchical clustering analysis of metabolites from kimchi broths. A heatmap was constructed for metabolites that showed significant mean differences (ANOVA, *p* < 0.05) based on Euclidean distance and Ward clustering algorithm. (**B**–**D**); Pathway over-representation analysis. The analysis was performed on the metabolites of each cluster.

Untargeted metabolic profiling was performed to examine whether the distinctive modification in metabolome co-occurred according to the different LED treatments. A total of 105 metabolites were structurally annotated and classified into nine groups based on the HMDB chemical ontology (Figure 2). The representative superclasses were as follows: organic acids and derivatives (25.71%), lipids and lipid-like molecules (22.86%), and organoheterocyclic compounds (20%) (Figure 2). Overall, differences in metabolite abundances (top 50 metabolites based on *p*-values from ANOVA) were characterized using hierarchical clustering analysis (HCA) (Figure 3A). Three clusters were identified with a distinctive abundance pattern. Metabolites in cluster-1 were characterized by relative enrichment in BLED-treated group and lowest levels in the initial condition. Metabolites in cluster-2 included compounds that were depleted following the 72 h incubation, regardless of the treatment. Metabolites in cluster-3 showed significantly lower abundances in BLED-treated group than other groups, whereas the metabolite contents were relatively higher in RLED-, GLED-, and no-LED-treated groups than in the BLED-treated group. Subsequent pathway over-representation analysis indicated the metabolic specificity for each cluster. In particular, cluster-3 showed significant enrichment in flavone–flavonol biosynthesis (FDR = 0.007) and flavonoid biosynthesis (FDR = 0.034) (Figure 3D), whereas moderate levels of metabolic reprogramming were found in pantothenate-CoA biosynthesis (cluster-1) and purine metabolism (cluster-2) (Figure 3B,C).

The metabolomic phenotype was further evaluated using unsupervised multivariate statistics, PCA. Twenty-two metabolites that were significantly different among the groups (ANOVA, *p* < 0.05) were used to perform the PCA analysis. The resultant score plot showed the distinctive profiles according to the treatment (R2X = 0.987, Q2 = 0.754). Apart from the initial condition, the BLED-treated group presented the most distinctive separation from the other groups (RLED, GLED, and dark condition) along with principal component 1 (PC1) and PC2 (a total variance explained: 84.5%) (Supplementary Figure S1A-PCA). Supervised multivariate statistics, OPLS-DA, recapitulated the group-wise separation validated by the high levels of R2Y (0.795) and Q2 (0.557) (Supplementary Figure S1C-OPLS-DA). 

**Figure 4:**
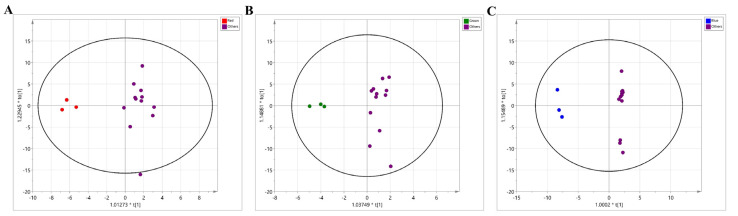
Multivariate statistical analysis of metabolic profiles of LED-irradiated kimchi broths. Orthogonal projection to latent structures-discriminant analysis (OPLS-DA) for pair-wise comparison between red-LED groups vs. other groups (R2Y = 0.930, Q2 = 0.445) (**A**), green-LED groups vs. other groups (R2Y = 0.925, Q2 = 0.368) (**B**), and blue-LED groups vs. other groups (R2Y = 0.997, Q2 = 0.896) (**C**).

Subsequently, we investigated the key metabolic features of each LED treatment. Pair-wise comparisons were conducted between individual group and other groups (e.g., RLED vs. all other groups) based on the OPLS-DA model of all 105 metabolites. The OPLS-DA model for the RLED group showed a high prediction level (R2Y = 0.930, Q2 = 0.445) (Figure 4A), and the top 10 metabolites based on VIP scores were as follows: 6-methylquinoline, isorhamnetin, 9,10-dihydroxy-12Z-octadecenoic acid, luteolin, 9-Oxo-10(E),12(E)-octadecadienoic acid, N-acetylsphingosine, nonivamide, skatole, serotonin, and capsaicin. Among them, four metabolites (VIP scores > 1.5) showed exclusively higher abundances in the RLED-treated group than in the other groups, including 6-methylquinoline, isorhamnetin, 9,10-dihydroxy-12Z-octadecenoic acid, and luteolin. Other metabolites were co-abundant or marginally different compared to those of the other groups. Nonivamide, capsaicin, and skatole were co-enriched in the RLED-treated group and dark control, whereas serotonin was co-abundant in RLED- and GLED-treated groups and the dark control. A total amount of phenol compounds in orange were found at increased levels by postharvest RLED-treatment [38]. The enrichment may be attributed to the activation of key enzymes in the phenylpropanoid pathway by LED treatment [39].

**Figure 5:**
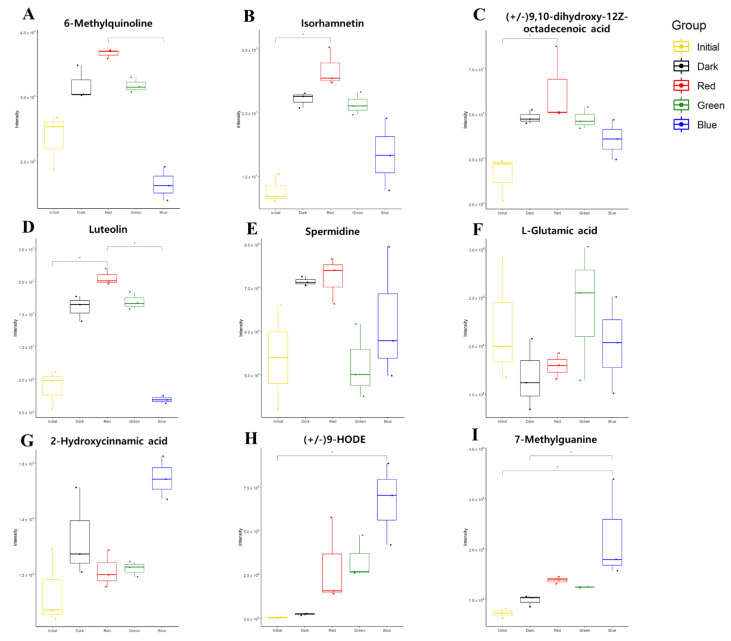
The relative abundances of the metabolites with characteristic alteration identified based on the OPLS-DA model. The abundances (ion intensities) are shown as box-whisker plot. *p* value is estimated based on Kruskal–Wallis test and Dunn’s test adjusted by Benjamini–Hochberg correction. * indicates *p* < 0.05. (**A**–**D**) are metabolites significantly altered in RLED group. (**E**,**F**) are metabolites significantly altered in GLED group. (**G**–**I**) are metabolites significantly altered in BLED group.

Likewise, the discriminant model for the GLED-treated group showed an acceptable level of the prediction quality (R2Y = 0.925, Q2 = 0.368) (Figure 4B). The GLED-treated group was characterized by a marginal increase in spermidine content and the lowest glutamate content, which was equivalent to that of the initial condition. The metabolites, which were top-ranked for the BLED-treated group, were characterized by significantly lower levels than those of the other groups, including quercetin, quercitrin, sinapinic acid, chlorogenic acid, rutin, and 6-methylquinoline (VIP > 1.5) (Figure 4C). The decreased flavonoids by BLED (e.g., quercetin and rutin [39]) were also reported in a previous study, in which 19 structural genes involved in flavonoid synthesis were downregulated in high-intensity blue LED light on growing plant species [40]. In contrast, 2-hydroxycinnamic acid, 9-HODE, and 7-methylguanine were at marginally higher levels than those in the other groups. Among the metabolites, 9-HODE level showed the most specific upregulation in all LED-treated groups compared to levels in the initial and dark conditions (Figure 5H). The compound metabolized from linoleic acid has been found at significantly lower levels in colorectal cancer (CRC) patients [41] and may be associated with the activation of peroxisome proliferator-activated receptor gamma (PPARγ) [42,43]. PPARγ shows anticancer activity by suppressing cell proliferation and inducing cell apoptosis [44,45].

### 2.3. Cellular Toxicity of Kimchi Broth Extracts Treated with LEDs

**Figure 6:**
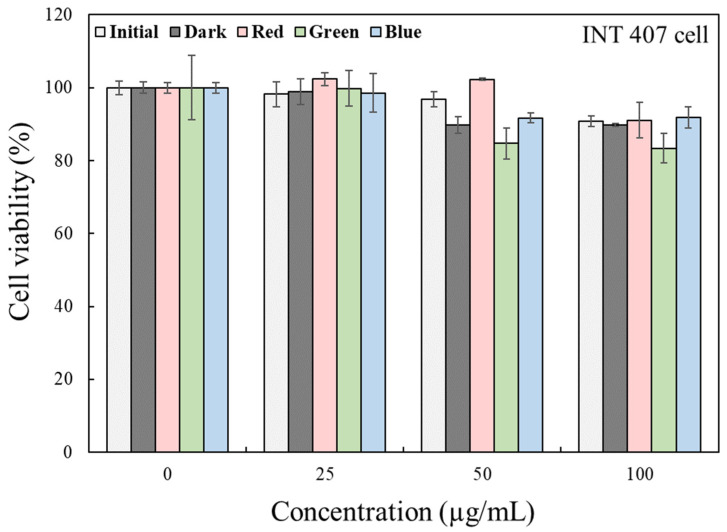
Effects of 3 LED-irradiated kimchi broths on viability of the INT 407 cell. The LED-irradiated kimchi broths were extracted and evaluated for cell viability. Cells were treated with LED-irradiated kimchi extracts (0–100 µg/mL). After 24 h of incubation, viable cells were analyzed using MTT assay.

Kimchi broths that underwent LED treatment and were kept in the dark (for 72 h) were extracted and evaluated for cytotoxic effects in human intestinal cell line (INT-407 cell). There was no significant difference in the cell viability among the extracts across all concentrations tested (Kruskal–Wallis U-test, *p* > 0.05) (Figure 6). In the present study, to maximize the effect of LED treatment on kimchi, for a rather long time (72 h), the changes in microbiota and metabolites in kimchi broth were analyzed following the LED treatment. When applied during processing or storage, the LED irradiation method is considered to be an intermittent treatment or a short-term irradiation method. In the present study, instead of using the PDT method to artificially add photo-sensitizing agents to induce changes in cell proliferation and cell survival, the LED–photobiomodulation reaction in kimchi broth was observed during kimchi fermentation under LED treatments. The PDT actions could simultaneously be induced with PBM by the presence of anthocyanins, carotenoids, and chlorophylls that act as a natural photosensitizer in the kimchi broth [3]. That is, the PDT action can be used to control food spoilage bacteria during fermentation, and the PBM action is expected to be usefully used by modulating microbiota composition during fermentation process without affecting cell viability. Thus, considering the LED treatment time, an in vitro cell cytotoxicity test demonstrated the safety and efficacy of these LEDs for the modulation of fermentation in kimchi processing and storage.

## 3. Materials and Methods

### 3.1. Sample Preparation

For sample preparation, red Chinese cabbage kimchi with red pepper, which had passed 1–2 days from date of manufacture, was purchased at a local retail store and stored at 4 °C prior to experiments. Kimchi (100 g of solid and liquid material) was weighed in a sterile workbench (HB-402; Hanbaek Co., Ltd., Bucheon, Korea), placed in a sterile bag (19 × 30 cm, Nasco WHIRL-PAK^®^, Fort Atkinson, WI, USA), and pulverized for 3 min using a blender (Digital LED Embo stomacher, BNF Korea Co., Ltd., Kimpo, Korea). The blended sample was centrifuged (10,000× *g*, 5 min) and the supernatant was transferred to a 15-mL conical tube. The kimchi broth sample (3 mL) was transferred to a sterile Petri dish (30 mm diameter) 3 times for each group (red-LED (RLED), green-LED (GLED), blue-LED (BLED), and dark condition) making 3 replicate samples. Although the volume applied in this study was relatively small compared to the one from typical kimchi (e.g., a home-made one), considering that kimchi samples are produced commercially at a small scale (approximately 50~100 mL per individual unit), the samples are representative within our current experimental scope.

### 3.2. Light Emitting Diode (LED) Light Chamber Setup

**Figure 7:**
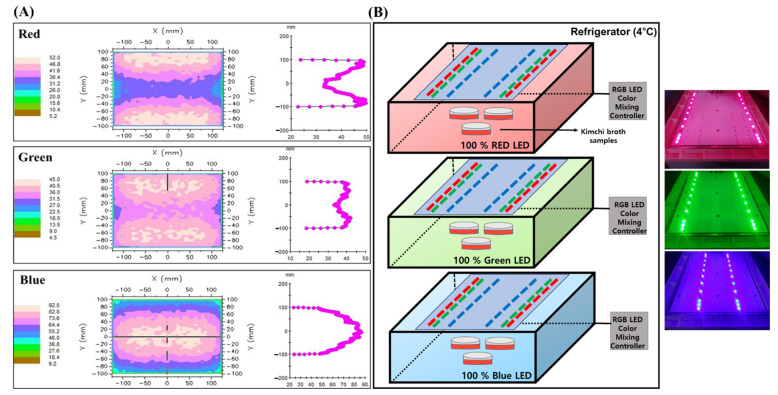
(**A**) Simulated irradiation patterns in LED light chamber at 10 mm distance. The inside dimensions of LED chambers were 25 × 20 × 10 cm (L × W × H). The distribution uniformity of the LED light systems is evaluated by optical analysis software packages, Light Tools (Synopsys Inc., Mountain View, CA, USA). (**B**) Schematic diagram of LED treatments.

LED treatment on kimchi broth was performed using a customized device manufactured by ESLEDs co., Ltd. (Seoul, Korea). The device was assembled for three different LED lights (Red; 60 mA, 1.90 V, Green; 60 mA, 2.79 V, Blue; 60 mA, 2.86 V). Luminous intensity was measured by Multi-LED light meter (TM-209M, Tenmars electronics CO., Ltd., Taipei, Taiwan). Wavelengths of 660 ± 5, 525 ± 5, and 450 ± 5 nm were applied for red-LED (RLED), green-LED (GLED), and blue-LED (BLED), respectively. Emission distance was set to 10 cm and intensity was kept constant. Density distribution pattern was analyzed by optical analysis software packages, Light Tools (Synopsys Inc., Mountain View, CA, USA). Figure 7A shows the results obtained by measuring the uniformity distributions of the LED light systems.

### 3.3. LED Treatment

Figure 7B shows that three dishes of Kimchi broth samples (3 mL) were placed below the light source in each LED chamber. The distance was adjusted between the sample and the LED light source (10 cm). After the plates were sealed by parafilm, samples in three individual plates (triplicate) were placed in each of the 3 LED light sources (Red, Green, and Blue) and then, the broths were incubated with LED-treatment at 4 °C for 72 h. Samples were incubated at dark condition as an additional control.

### 3.4. DNA Extraction, Polymerase Chain Reaction Amplification, Quantification, and Metagenome Sequencing of 16S Ribosomal RNA

DNA was extracted using a DNeasy PowerSoil Kit (Qiagen, Hilden, Germany) according to the manufacturer’s instructions. DNA quantity and quality were measured using the NanoDrop 1000 spectrophotometer (Thermo Fisher Scientific, Wilmington, DE, USA). The sequencing libraries is prepared according to the Illumina 16S Metagenomic Sequencing Library protocols to amplify the V3 and V4 region. The input gDNA 2 ng was PCR amplified with 5x reaction buffer, 1 mM of dNTP mix, 500 nM each of the universal F/R PCR primer, and Herculase II fusion DNA polymerase (Agilent Technologies, Santa Clara, CA). The cycle condition for 1st PCR was 3 min at 95 °C for heat activation, and 25 cycles of 30 sec at 95 °C, 30 sec at 55 °C, and 30 sec at 72 °C, followed by a 5-min final extension at 72 °C. The universal primer pair with Illumina adapter overhang sequences used for the first amplifications were as follows: V3-F: 5′-TCGTCGGCAGCGTCAGATGTGTATAAGAGACAGCCTACGGGNGGCWGCAG-3′, V4-R: 5′- GTCTCGTGGGCTCGGAGATGTGTATAAGAGACAGGACTACHVGGGTATCTAATCC-3′. The 1st PCR product was purified with AMPure beads (Agencourt Bioscience, Beverly, MA, USA). Following purification, the 2 µL of 1st PCR product was PCR amplified for final library construction containing the index using NexteraXT Indexed Primer. The cycle condition for 2nd PCR was same as the 1st PCR condition except for 10 cycles. The PCR product was purified with AMPure beads. The final purified product is then quantified using qPCR according to the qPCR Quantification Protocol Guide (KAPA Library Quantificatoin kits for IlluminaSequecing platforms) and qualified using the TapeStation D1000 ScreenTape (Agilent Technologies, Waldbronn, Germany). The paired-end (2 × 300 bp) sequencing was performed by the Macrogen using the MiSeq™ platform (Illumina, San Diego, CA, USA).

### 3.5. Metagenomics Sequence Analysis

The illumine paired-end data as 2 FASTQ files were uploaded to the EzBioCloud 16S-based MTP app (ChunLab Inc., Seoul, Korea) to check the data quality. EzBiocloud Microbiome Taxonomic Profile (MTP) pipeline was employed for diversity estimation using PKSSU4.0 version database and Open reference UCLUST_MC2 for OTUs picking at 97% cut-off. The MTP app was used to detect and filter out sequences of low quality with regard to read length (<100 bp or >2000 bp) and averaged Q values less than 25. Microbial richness was measured by Chao1 and the number of OTUs found in the microbiome taxonomic profile (MTP) index. The alpha diversity indices are also applied, including the ACE (abundance coverage estimator) [46], Chao1 [47], Jackknife [48], Shannon [49], and Simpson [50] indices. The indices include statistical estimates of species richness (Ace, Chao, Jackknife), and estimates of species evenness (Shannon, Simpson).

### 3.6. Metabolomic Analysis of Kimchi Extracts

All experiments were performed in triplicate and analyzed in random order. Kimchi broth (100 µL) was extracted by organic solvent mixture (1 mL, methanol:isopropanol:water, 3:3:2, *v*/*v*/*v*) for metabolomic profiling. After 5-min sonication and centrifugation, aliquot (800 µL) was transferred to a new vial and concentrated to complete dryness using a speed vacuum concentrator (SCANVAC, Seoul, Korea). The dried sample was re-constituted with 100 µL of 0.03% acetic acid in D.W for mass-spectrometric analysis. Chromatography was performed on a Waters Acquity UPLC BEH C18 column (2.1 mm × 150 mm, 1.7 μm) controlled by an Ultimate-3000 UPLC system (Thermo Fisher Scientific, Waltham, MA, USA) [51]. The mobile phase consists of solvent A (H_2_O with 0.03% acetic acid) and B (acetonitrile with 0.03% acetic acid). Flow rate was set to 300 µL/min and gradient was programmed as follows: (B): 0–0.1 min, 0.5%; 0.1–10.0 min, 0.5–80%; 10.0–10.1 min, 80–99.5%; 12.0–12.1 min, 99.5–0.5%; 12.1–15 min, 0.5%. Metabolite profiling was conducted using liquid chromatography based Orbitrap mass spectrometry in a Q-Exactive Plus instrument (Thermo Fisher Scientific, Waltham, MA, USA). Full scan mode (MS1) was set to the range from 100 to 1500 Da (resolution: 70,000 FWHM) for both positive and negative ions. Tandem mass spectra (MS/MS) were collected for the top 5 most intense from the pooled samples in a data-dependent manner. Compound identification and quantitation were done using a Compound Discoverer (ver 3.0, Thermo Fisher Scientific, San Jose, CA, USA). Criteria for compound identification were set to as follows: (1) max retention time shift: 1 min for peak alignment; (2) MS1 tolerance: 5 ppm, MS/MS tolerance: 10 ppm, assignment threshold: 70% similarity against mzCloud. Relative intensity for each metabolite was calculated based on peak area.

### 3.7. Statistical Analysis

The data matrix was normalized using total ion intensity for further statistical analysis. Treemap was generated using Microsoft Excel (Microsoft, Seattle, WA, USA) based on chemical taxonomy (superclass and class) from the Human Metabolome Database (HMDB). Multivariate statistical analysis, including PCA and OPLS-DA, was done using SIMCA 15 (Umetrics AB, Umea, Sweden). Pathway over-representation analysis was done based on centrality and the hypergeometric test in the web server, MetaboAnalyst (https://www.metaboanalyst.ca/ accessed on 12 April 2021). A heatmap was constructed based on the Euclidean distance and Ward clustering algorithm in the MetaboAnalyst server. Box and whisker plots were generated using ggpubr function in ggplot2 packages (R version 4.0.2 and RStudio version 1.3.959).

### 3.8. Cell Viability Assay

For cell viability assay, the kimchi broth (1 mL; described in Section 3.1) was extracted three times by ethyl acetate (1 mL). The extracts were centrifuged under the same conditions (10,000× *g* for 5 min) and the pooled extracts were evaporated at 60 °C. MTT assay was applied to evaluate cell cytotoxicity based on INT 407 immortalized intestinal cell (ATCC CCL-6) purchased from ATCC (American Type Culture Collection, Manassas, VA, USA). Cells (1.5 × 10^4^ per well) were plated in 96-well plates and incubated for 24 h at 37 °C. After the dose-dependent treatment with the samples (kimchi extracts) for 24 h, MTT (0.5 mg/mL, 100 µL) was added and the cells were further incubated in CO^2^ chamber for 30 min. The absorbance was measured at 550 nm using microplate reader (SpectraMax M3, Molecular Devices, Sunnyvale, CA, USA).

## 4. Conclusions

In the current study, we investigated the effects of different wavelengths of LED on kimchi broth using multiomics (metataxonomics and metabolomics) coupled to functional assays. Metataxonomic profiles showed fermentation and LED treatment-dependent changes in the microbial composition. GLED showed the strongest influence on the assembly of *Weissella* and *Lactobacillus,* the two major strains observed in kimchi fermentation. Subsequently, we explored the unique metabolic phenotype and identified bioactive compounds using the LED treatment. Thereby, we propose that LED treatment influences microbial composition and consequently reprograms the microbial metabolome, which may confer the putative bioactivity. Alternatively, it is also plausible that the metabolites attributed to the functionality are converted by the photodynamic activity of LED emission itself or through their interaction. The results emphasize the potential of LED application to modulation of microbial composition and to production of beneficial compounds particularly in fermentation food.

## Data Availability

All data, tables and figures in this manuscript are original.

## Supplementary Materials

The following are available online at https://www.mdpi.com/article/10.3390/metabo11080472/s1, Table S1: Changes in bacterial community richness and diversity indices in LED-treated red pepper kimchi, Figure S1: Multivariate statistical analysis of metabolic profiles of LED-irradiated kimchi broths.
