# Supplementary material for: Metagenomic, Metabolomic, and Functional Evaluation of Kimchi Broth Treated with Light-Emitting Diodes (LEDs)
Source: Metabolites. 2021 Jul 22;11(8):472. doi: 10.3390/metabo11080472 (PMC8401942; doi:10.3390/metabo11080472)
Supplement: Supplementary file 1 [file metabolites-11-00472-s001.zip › metabolites-1293187-supplementary.pdf]

Article

Metagenomic, Metabolomic, and Functional Evaluation of Kimchi Broth Treated with Light-Emitting Diodes (LEDs)

Yeong Ji Oh [1,*,†], Ye Rin Park [1,†], Jungil Hong [2] and Do Yup Lee [1,*]

[1] Department of Food and Animal Biotechnology, Research Institute for Agriculture and Life Sciences, Center for Food and Bioconvergence, Seoul National University, Seoul 08826, Republic of Korea; yerinpark@snu.ac.kr

[2] Division of Applied Food System, College of Natural Science, Seoul Women's University, 621 Hwarangro, Nowon-gu, Seoul 01797, Republic of Korea; hjil@swu.ac.kr

* Correspondence: rome73@snu.ac.kr (D.Y.L.); anabaena@snu.ac.kr (Y.J.O.)

† These authors contributed equally to this work.

Supplementary Materials

Table S1. Changes in bacterial community richness and diversity indices in LED-treated red pepper kimchi.

Sample name	Target reads	[1]OTUs	[2]ACE	[3]Chao1	[4]Jackknife	[5]Shannon	[6]Simpson	[7]Good's coverage of library (%)
Initial	86767	178	252.31	226.00	242.51	1.01	0.45	99.93
Dark	73832	159	215.14	198.37	212.19	0.76	0.65	99.93
Red	91661	137	207.63	178.13	188.62	0.70	0.69	99.94
Green	86547	140	180.56	165.80	183.00	0.60	0.74	99.95
Blue	83248	149	221.04	212.21	218.29	0.65	0.71	99.93

[1] OTUs: Operational Taxonomic Unit (OTU) is a group of sequences clustered by sequence similarity.

[2] ACE: ACE (Abundance-based Coverage Estimator) is an indicator of species richness (total number of species in a sample) that is sensitive to rare OTUs (singletons and doubletons)

[3] Chao1: Chao1 is an indicator of species richness (total number of species in a sample) that is sensitive to rare OTUs (singletons and doubletons).

[4] Jacknife: Jackknife is an indicator of species richness (total number of species in a sample) that is sensitive to rare OTUs (singletons and doubletons) as well as to abundant OTUs (tripletons and more).

[5] Shannon: The Shannon index takes into account the number and evenness of species.

[6] Simpson: Simpson is an indicator of species evenness (proportional distribution of the number of each species in a sample) that displays the probability that two randomly selected sequences are of the same species.

[7] Good's coverage of library (%): number of sequencing reads used for analysis represents the actual species population of the sample. The value can range from 0 to 100%, with 100% indicating a complete sampling of species, meaning that additional sequencing is unlikely to find any more new species.

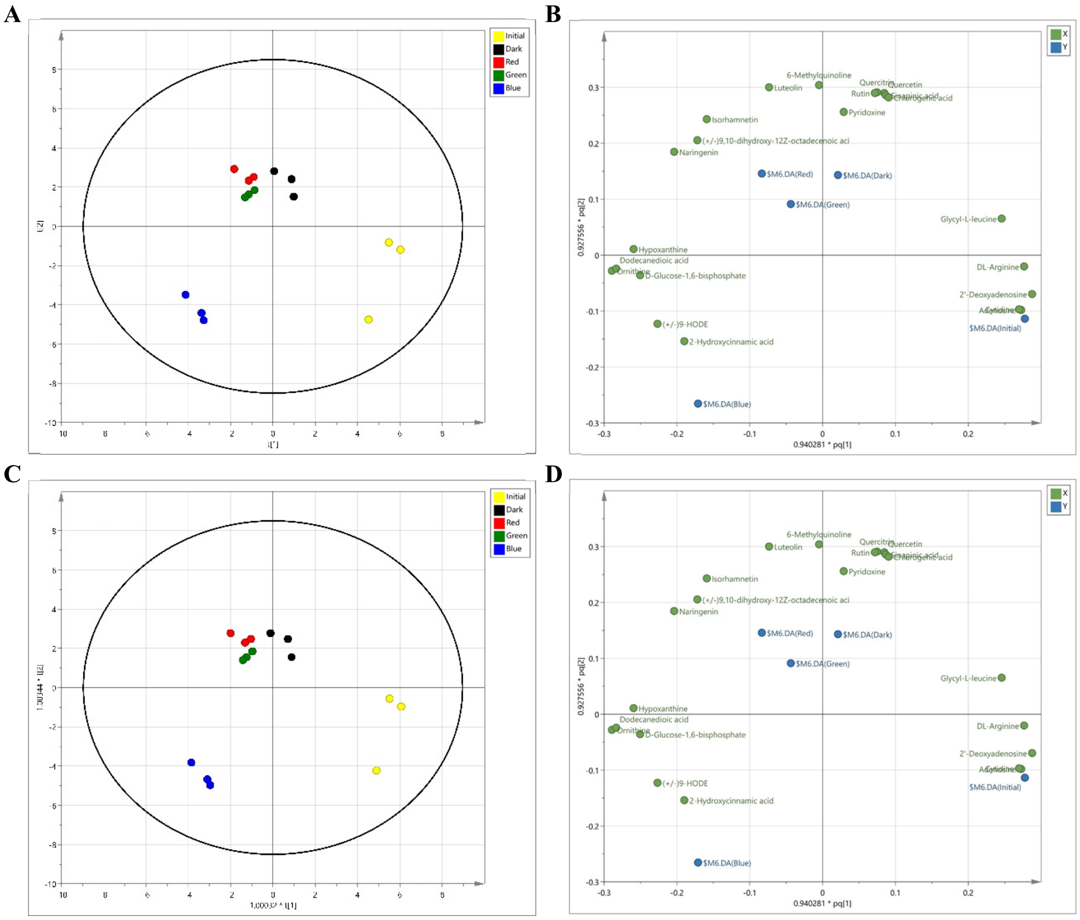

Figure S1. Multivariate statistical analysis of metabolic profiles of LED-irradiated kimchi broths. 22 metabolites that were significantly different among the groups (ANOVA, $p < 0.05$) were used to perform the multivariate analysis. (A) Score plot of the kimchi extracts based on principal component analysis (PCA). The variance is most explained by t1 vector (44.5%). 2 components are needed to reach 84.5 % of overall variance. (B) Loading plot based on PCA. (C) Score plot of the kimchi extract based on orthogonal projection to latent structures-discriminant analysis (OPLS-DA). The variance is explained by PC1 and PC2 at 24.6 % and 23.0 % for each component. 4 components are needed to reach 82.4 % of overall variance. (D) Loading plot based on OPLS-DA.
